# Genotypic analysis of drug-resistant tuberculosis in Ghana: Insights into pre-XDR and XDR-TB

**DOI:** 10.1371/journal.pone.0323527

**Published:** 2025-05-20

**Authors:** Stephen Ofori Yirenkyi, Osmanu Taufik, Roger Laryea, Amanquador Acquah-Keelson, Japheth Awuletey Opintan, Gloria Ivy Mensah

**Affiliations:** 1 Laboratory Department, Eastern Regional Hospital, Koforidua, Eastern Region, Ghana; 2 Department of Medical Microbiology, University of Ghana Medical School, University of Ghana, Accra, Ghana; 3 Department of Medical Laboratory Sciences, School of Allied Health Sciences, University of Health and Allied Sciences, Ho, Ghana; 4 Department of Bacteriology, Noguchi Memorial Institute for Medical Research, University of Ghana, Accra, Ghana; Rutgers Biomedical and Health Sciences, UNITED STATES OF AMERICA

## Abstract

**Background:**

The emergence of Extensively Drug Resistant (XDR) and Pre-extensively drug resistant (Pre-XDR) tuberculosis (TB) threatens the management of multidrug-resistant tuberculosis (MDR) patients and impacts negatively on TB control programs, especially in developing countries like Ghana. The first case of XDR-TB in Ghana was reported in 2018. There is however inadequate data on the burden of XDR-TB and pre-XDR-TB and their associated resistant mutations in Ghana. The study sought to provide baseline data on the burden of pre-XDR-TB and XDR-TB among MDR TB cases in Ghana. It also determined the mutations responsible for pre-XDR/ XDR-TB, for clinical and programmatic management of pre-XDR/ XDR-TB in Ghana.

**Methods:**

One hundred and seventy-one (171) archived clinical MDR isolates obtained from TB patients across Ghana between January 2016 and December 2020 were retrieved. The isolates were retested to confirm their phenotypic and genotypic susceptibility to the first and second-line anti-TB drugs using the BACTEC MGIT system and Genotype MTBDR*plus*, MTBDR*sl*, line probe assays respectively.

**Results:**

Most of the 171 isolates came from 7 regions; the highest (39.5%) from Eastern, followed by Greater Accra region (19.8%). Most of the isolates were from male TB patients (78.9%). Of the 171 archived isolates, 81 (47.4%) were confirmed to be MDR, 6 (7.4%) were Pre-XDR-TB but no XDR-TB was detected. The *katG* S315T1 (33, 73.3%) and *rpoB* S531L (31, 42.5%) were the predominant mutations observed among isoniazid and rifampicin resistant isolates respectively. Many of the mutations and amino acid changes that caused pre-XDR-TB were *gyrAWT3 + gyrAMUT3A* and *gyrAMUT3A* (D94A) (50%) for fluoroquinolone. The other detected mutations with their amino acid changes were *gyrA* MUT1 (A90V), *gyrA*WT3 + *gyrA* MUT3C (D94G) and *gyrA* MUT2 (S91P) (16.7%) for fluoroquinolones and *rrWT2* (position 1484) (33.3%) and *rrs* MUT2 (G1484T) (16.7%) for aminoglycosides.

**Conclusion:**

The predominant mutations associated with pre-XDR-TB were D94A and C1402T for fluoroquinolone and aminoglycosides resistance respectively. The proportion of pre-XDR-TB among MDR-TB patients in Ghana was 7.4%; however, no XDR-TB was detected. A sustained surveillance of pre-XDR-TB and XDR-TB is recommended.

## Introduction

Tuberculosis (TB) continues to be a serious health problem with a huge public health burden globally (10.8 million new cases in 2023), especially in low and middle-income countries, despite concerted efforts to control it [1,2]. In Ghana, where DOTS (Directly Observed Treatment Short Course) is still used to combat tuberculosis, the infection remains one of the most common communicable diseases [3].

Globally, TB control programs are finding it increasingly difficult to control and manage TB cases for several reasons. The spread of resistant strains, particularly multidrug-resistant (MDR) TB strains challenge national control efforts, and increases the burden of this contagious deadly infection [4]. MDR-TB poses a serious threat to global TB control and burdens developing nations with costly and toxic therapies, that worsens the tuberculosis epidemic [5]. The emergence and spread of drug resistance within the last decade and the progression from MDR-TB to pre-extensively drug resistant (pre-XDR) and extensively drug resistant tuberculosis (XDR-TB) has complicated TB management [6]. Pre-XDR-TB is defined as TB *caused by MTBC strain that fulfils the definition of MDR/RR-TB and is also resistant to any fluoroquinolone (FQ),* and extensively drug-resistant (XDR)-TB denotes *TB caused by MTBC strain that fulfils the definition of MDR/RR-TB with additional resistance to any FQ [ofloxacin, levofloxacin or moxifloxacin] and at least one of bedaquiline (BDQ) and linezolid (LZD*) [7,8]. XDR-TB, a considerably more difficult-to-treat type of MDR-TB, is spreading, from initially being recorded in 46 countries in 2008 [8] to 77 countries in 2017, with a treatment success rate of only 34% [9]. By the end of 2023, 100 countries worldwide had reported at least one case of XDR-TB [2].

Currently, molecular techniques provide accurate TB diagnosis and evaluate the resistance status of the bacteria based on the association between mutations affecting the function and expression of chromosome-encoded targets and resistance to anti-tuberculosis drugs [10]. In the last decade, resistance to rifampicin (RIF), isoniazid (INH), FQ and second-line injectable anti-TB drugs have been well documented in several studies [11–13]. RIF resistance is mainly due to point mutations in the *rpoB* gene while mutations in *katG*, *ahpC* and *inhA* genes account for INH resistance [10,14,15]. Primarily, FQ resistance results from point mutations in the genes encoding the two DNA subunits, *gyrA* and *gyrB* [16], while majority of the mutations causing FQ resistance are concentrated in a brief region of the *gyrA* gene known as the Quinolone Resistance Determining Region (QRDR) [16,17]. Mutations in the *rrs* genes are frequently known to confer resistance to injectable drugs, however, mutations in the *tlyA* gene and *eis* gene have also been reported [15,18].

Estimating the burden of XDR-TB and pre-XDR-TB among MDR-TB as well as identifying the drug resistance-conferring mutations in *M. tuberculosis* is important for targeted TB control especially in endemic countries like Ghana. A recent study in Ghana identified several pre-XDR TB cases among difficult-to-treat tuberculosis patients [19]. The present study focused on identifying drug resistance-conferring mutations and patterns of the mutations among MDR-TB patients in Ghana using archived clinical MDR isolates obtained from TB patients across Ghana between January 2016 and December 2020.

## Materials and methods

### Study design

This study employed a cross-sectional experimental design. Archived clinical MDR-TB strains, isolated from TB patients between January 2016 to December 2020 within 10 administrative regions of Ghana were used.

### Study site and sample collection

Chest Clinic TB Laboratory of the Korle Bu Teaching Hospital, Accra, and the TB Laboratory of the Eastern Regional Hospital, Koforidua are the two main laboratory networks in the diagnosis of Drug Resistance TB within the Ghana Health Service. One hundred and seventy-one (171) archived clinical isolates from MDR-TB patients, stored in Tryptophan soy broth-glycerol at -20°C at these laboratories were obtained and analyzed at the TB lab of the Eastern Regional Hospital, Koforidua.

### Laboratory analysis

#### Isolates recovery.

The isolates were sub-cultured in a liquid medium (modified Middlebrook 7H9) using BACTEC MGIT 960™. Positive culture tubes were checked for mycobacterial growth by microscopic examination of Ziel Nielsen (ZN) stained smears from the broth culture, and confirmed using MGIT TBc Identification kit to exclude Non tuberculous Mycobacteria (NTMs).

#### Phenotypic drug susceptibility testing.

Pure colonies of *M. tuberculosis* complex (MTBC) were tested for their antimicrobial susceptibility to the first-line anti-TB drugs (Streptomycin, Isoniazid, Rifampicin and Ethambutol) at critical concentrations by the broth dilution method using BACTEC™ MGIT™ SIRE® kit on the BACTEC™ MGIT™ system from BD. The instrument and kit manufacturer instructions were observed. The final drug concentrations in the test bottles were 1.00µg/ml for Streptomycin, 0.10µg/ml for INH, 1.00µg/ml for Rifampicin and 5.00µg/ml for Ethambutol.

Automated reading of the DST on BACTEC™ MGIT™ 960 system was done after 13–14 days of loading. Results were interpreted as follows; the drug-containing tube with a Growth Unit (GU) ≥100 was resistant, while GU of a drug-containing tube ≤100 was susceptible. Tested-Positive External Quality Control (EQA) panel strains were included as quality controls.

Isolates with confirmed resistance to RIF and INH were included in the genotypic susceptibility testing of second-line anti-TB drugs (Fluoroquinolones and aminoglycosides) using the Line Probe Assay (LPA) from Hain Lifescience (Nehren, Germany).

#### DNA extraction and PCR amplification.

DNA was extracted from the *M. tuberculosis* isolates by heat-alkaline method using the GenoLyse extraction kit Version 2.0 (Hain Life Science, Germany). The manufacturer’s instructions were strictly followed. The DNA extract was used for the PCR procedure and stored at -20°C for further analysis.

The Genotype MTBDR*plus* VER 2.0^®^ and Genotype MTBDR*sl* VER 2.0^®^ LPA, a multiplex PCR procedure was used for the DNA amplification for the first line anti-TB drugs (RIF and INH) and second line anti-TB drugs (FQs and AMGs) respectively, following manufacturer’s instructions.

#### Hybridization.

After PCR, the GT Blot 48^®^ instrument was used for the line probe assay: a reverse hybridization process. The amplification products of biotin-labelled dsDNA amplicons of the genes of interest were denatured using NaOH denaturation solution (DEN) to break the hydrogen bonds between the paired nucleotides.

**Fig 1:**
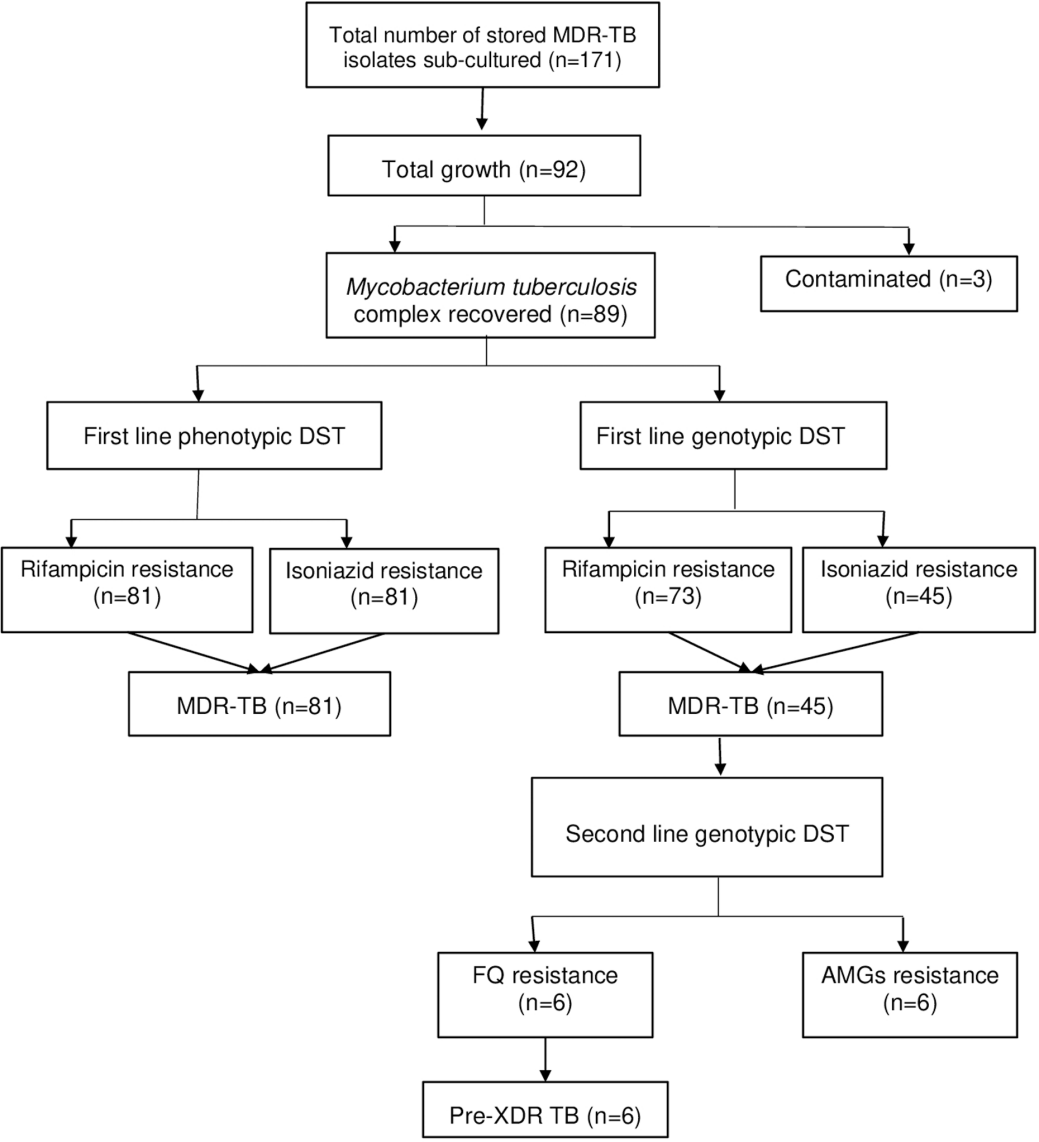
Flowchart showing confirmed MDR and pre-XDR strains of *M. tuberculosis* from archived isolates.

Deoxyribonucleic acid (DNA) strip (labelled with sample ID) with probes (reaction zones) of unlabelled complementary sequences immobilized as bands on a positively charged nitrocellulose membrane strips were suspended in the amplification product and DEN mixture. Various steps of addition of hybridization, stringent, rinse and substrate solutions were followed according to the manufacturer’s instructions. The specific regions of genes (wild-type or mutant) present in the heterogeneous mixture of the target ssDNA were detected by visual inspection of the strips. Fig 1 shows the flow of work from subculture of the archived isolates to detection of Pre-XDR strains.

#### Statistical analysis.

Data was entered into the Microsoft office excel 2016 spreadsheet. Descriptive and inferential analysis were done using IBM Statistical Package for the Social Sciences (SPSS Inc., Chicago, USA) (www.spss.com) version 25 to estimate the rate of mutations of *rpoB*, *katG* and *inhA gyrA, gyrB, rrs* and *eis* genes in the categories of patients.

#### Quality assurance.

Quality check of MGIT reagents and all other reagents was done before use. *Mycobacterium tuberculosis* External quality control strains, obtained from Korle Bu Teaching Hospital Chest Clinic Laboratory, were included in the MGIT liquid culture procedures and the molecular procedures.

#### Ethical consideration.

Ethical clearance for this study was sought from and duly approved by the Ethical and Protocol Review Committee (EPRC) of the College of Health Sciences, University of Ghana.

## Results

### Demographics of patients

Of the 171 archived isolates, 135 (78.95%) were from male patients. The ages of the patients ranged from 5 to 84 years with a mean age of **42.92 ± 15.63.** The MDR-TB isolates were obtained from sputum of patients residing in 7 regions of Ghana, including Ashanti (36), Eastern (85), Central (12), Greater Accra (20), Volta (4), Western (12) and Upper West (2). (Table 1)

**Table 1:** Demographic details of TB patients from whom the isolates were obtained.

Parameter	Frequency (n = 171)	Percentage (%)
**Mean age**	**42.92 ± 15.63**	
**Gender**		
Male	135	78.95
Female	36	21.05
**Age group**		
<30	34	19.88
30-60	114	66.67
> 60	23	13.45
**Regions**		
Ashanti	36	21.05
Eastern	85	49.71
Central	12	7.02
Greater Accra	20	11.70
Volta	4	2.34
Western	12	7.02
Upper West	2	1.17

### Mutational patterns observed

**Fig 2:**
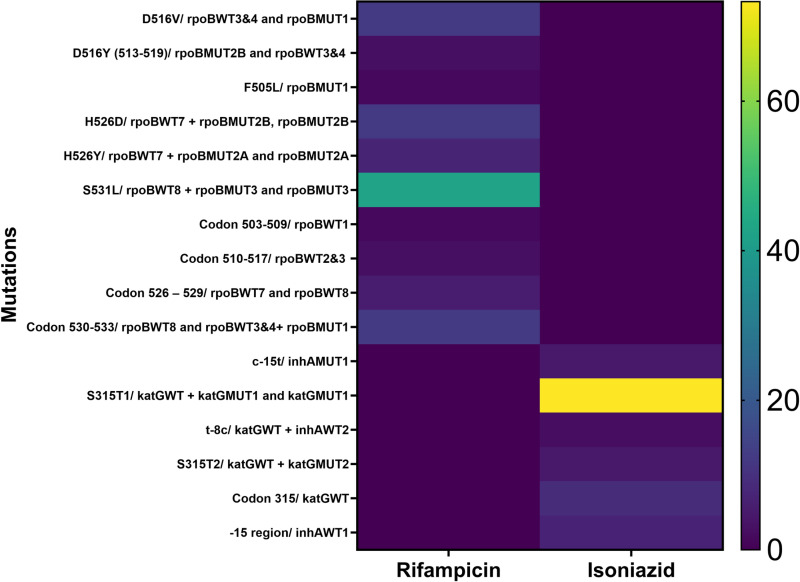
Heat Map of the Gene Mutation Profile of Samples.

Table 2 shows that among the RIF (*rpoB* gene) mutations, the commonly observed patterns were S531L (42.47%), D516V (12.33%), H526D (12.33%), Codon 530–533 (12.33%) and H526Y (6.85%). Of the 45 INH resistance mutations among the MDR-TB isolates by LPA, 39 (86.67%) were *katG* (high level resistance) and 5(11.11%) were i*nhA* (low level resistance). One (2.22%) was of both *katG* and *inhA* type mutations. The predominant *katG* mutation pattern was S315T1 (73.33%) while that of *inhA* was -15 region (6.67%). The gene mutation profiles with the highest frequency were S315T1/ katGWT + katGMUT1 and katGMUT1 (73.3%) for rifampicin and S531L/ rpoBWT8 + rpoBMUT3 and rpoBMUT3 (42.5%) for isoniazid (Fig 2). The other 14 gene mutation profiles each had a frequency of between 1 and 13%.

**Table 2:** First line drug mutation patterns observed among MDR-TB isolates using Genotype MTBDR*plus®.*

Drug	Mutation patterns	Gene mutation profile	Number of Samples (Percentage)
**RIF**	**D516V**	*rpoBWT3&4* and *rpoBMUT1*	9(12.3)
	**D516Y (513–519)**	*rpoBMUT2B* and *rpoBWT3&4*	2(2.7)
	**F505L**	*rpoBMUT1*	1(1.4)
	**H526D**	*rpoBWT7 *+ *rpoBMUT2B*, *rpoBMUT2B*	9(12.3)
	**H526Y**	*rpoBWT7 *+ *rpoBMUT2A* and *rpoBMUT2A*	5(6.9)
	**S531L**	*rpoBWT8* + *rpoBMUT3* and *rpoBMUT3*	31(42.5)
	**Codon 503–509**	*rpoBWT1*	1(1.4)
	**Codon 510–517**	*rpoBWT2&3*	2(2.7)
	**Codon 526–529**	*rpoBWT7* and *rpoBWT8*	4(5.5)
	**Codon 530–533**	*rpoBWT8* and *rpoBWT3&4* + *rpoBMUT1*	9(12.3)
**IHN**	**c-15t**	*inhAMUT1*	2(4.4)
	**S315T1**	*katGWT* + *katGMUT1* and *katGMUT1*	33(73.3)
	**t-8c**	*katGWT* *+ inhAWT2*	1(2.2)
	**S315T2**	*katGWT* + *katGMUT2*	2(4.4)
	**Codon 315**	*katGWT*	4(8.9)
	**-15 region**	*inhAWT1*	3(6.7)

Of the 12 second line drug (FQ and AMG) resistance mutations among the MDR-TB isolates 6(50%) were of *gyrA* mutations while the other half were *rrs* associated mutations. The predominant *gyrA* mutation pattern was D94A (26.67%) while that of *rrs* was C1402T (20%). (Table 3)

**Table 3:** Second line drug mutation patterns observed among MDR-TB using Genotype MTBDR *plus®.*

Drug	Mutation patterns	Gene mutation profile	Number of Samples (Percentage)
**FQ**	**A90V**	*gyrAMUT1*	1(16.7)
	**D94A**	*gyrAWT3* + *gyrAMUT3A* and *gyrAMUT3A*	3(50)
	**D94G**	*gyrAWT3* + *gyrAMUT3C*	1(16.7)
	S91P.	*gyrAMUT2*	1(16.7)
**AMG**	**C1402T**	*rrsWT1*	3(50)
	**G1484T**	*rrsMUT2*	1(16.7)
	**Position 1484**	*rrsWT2*	2(33.3)

### Observed MTBDR*plus* and MTBDR*sl* assays band patterns for first- and second-line anti-TB drugs respectively

Among the isolates resistant to at least one first-line anti-TB drug, the mutants were either *rpoB, inhA, katG,* and/or combinations of *katG* and *inhA*. There were 59 isolates with absence of *rpoB* wild type bands and 56 with presence of *rpoB* mutation bands among the RIF resistant samples. The most common RIF wild type band absent was *rpoBWT8* (61.02%) while the most common *rpoB* wild type mutation band present was *rpoBMUT3* (57.14%). The study also observed that among the INH resistant isolates, there were 3(8.57%) with absence of *inhA* wild type bands and 31(88.57%) with *katG* wild type bands absent. The most common wild type mutation band present among INH resistant isolates was *katGMUT2* (82.93%) Table 4.

**Table 4:** Observed MTBDR*plus* assay band patterns for first line anti-TB drugs.

Drug	Locus	WT band absent	%	Mutation bands present	%
**RIF**	*rpoB*	*rpoBWT1*	1(1.7)	*rpoBMUT1*	10(17.9)
		*rpoBWT2&3*	1(1.7)	*rpoBMUT2A*	5(8.9)
		*rpoBWT3&4*	12(20.3)	*rpoBMUT2B*	9(16.1)
		*rpoBWT7*	9(15.3)	*rpoBMUT3*	32(57.1)
		*rpoBWT8*	36(61.0)		
**INH**	*inhA*	*inhAWT1*	3(8.6)	*inhAMUT1*	3(7.3)
	*katG*	*katGWT*	31(88.6)	*inhAMUT3A*	1(2.4)
	*katG, inhA*	*katGWT, inhAWT2*	1(2.9)	*katGMUT1*	34(82.9)
				*katGMUT2*	3(7.3)

Among isolates resistant to at least one second-line anti-TB drug, the mutants were either *gyrA or rrs.* There were 3 isolates with absence of wild type *gyrA* bands and 6 with presence of *gyrA* mutation bands among the FQs resistant isolates. There were 6 isolates with absence of wild type *rrs* bands with one *rrs* mutation band present among the AMGs resistant isolates. The most common *gyrA* mutation band present was *gyrAMUT3A* (20.0%). (Table 5)

**Table 5:** Observed MTBDR assay band patterns for second line anti-TB drugs.

Drug	Locus	WT bands absent	%	Mutation bands present	%
**FQ**	***gyrA***	*gyrAWT3*	3 (20.0)	*gyrAMUT1*	1(6.7)
				*gyrAMUT2*	1(6.7)
				*gyrAMUT3A*	3(20.0)
				*gyrAMUT3B*	1(6.7)
**AMG**	***rrs***	*rrsWT1*	4(26.7)	*rrsMUT2*	1(6.7)
		*rrsWT2*	2(13.3)		

## Discussion

Public health initiatives to control tuberculosis continue to be threatened by the rising rates of rifampicin (RIF) and isoniazid (INH) resistance as well as MDR-TB [11]. MDR-TB has become a major public health concern in both industrialized and developing nations [20]. It is recommended that in all cases of TB, culture and drug susceptibility testing (DST) is done. This is because drug resistant *M. tuberculosis* poses a challenge to the use of standard regimens for TB therapy, particularly among patients who have already received treatment [21,22].

In this study, it was found that among the *rpoB* gene mutations, the most frequently observed mutation conferring RIF resistance was S531L (42.47%), followed by D516V (12.33%), H526D (12.3%) and H526Y (6.85%) indicating high resistance to rifampicin. Rifampicin binds to the β-subunit of the RNA polymerase and these mutations usually occur within the amino acid 507–533 regions of the *rpoB* region [23]. Our finding compares with previous reports in Africa [18–24] and China [25]. However, lower proportions have been reported from India [26]. Likewise, in this study, the most frequently observed mutation conferring INH resistance was S315T1 (73.33%). This is consistent with the earlier report of Addo et al., (2017) which recorded 77.8% S315T1 mutation among INH-resistant strains from a national population-based TB prevalence survey in Ghana.

The emergence and spread of XDR-TB and MDR-TB poses a serious risk to global health [27]. For this reason, the present study assessed the resistance of the MDR TB isolates to second line anti TB drugs and further determined the burden of pre-XDR and XDR-TB among MDR-TB patients. The study recorded a drug resistance rate of 7.4% for Fluoroquinolones (FQs) and 4.9% and 2.5% Aminoglycosides (AMGs) resistance and aminoglycosides resistance*(inferred*) respectively. Injectable second-line medications and FQs are both very powerful against MDR-TB. However, the treatment of MDR-TB will be more challenging if there is resistance to FQs or second-line injectable medicines. In light of this, diagnosing patients with pre-XDR-TB will enable clinicians to closely monitor these patients and halt the development of XDR-TB, which is more challenging to treat [28].

In this study, cases of pre-XDR-TB as per the new WHO definitions [29], which is MDR-TB with resistance to fluoroquinolones, was 7.4% among the MDR-TB isolates. Similar studies by Goyal et al., (2017) [30] in India and Shibabaw et al., (2020) [31] in Ethiopia reported 7.9% and 5.7% pre-XDR TB respectively while a more recent study in Ghana among difficult to treat TB patients also reported 7.4% [19]. However, compared with our findings, pre-XDR-TB has been found to be more common in China (66.4%), Cambodia (13.6%) and Brazil (15%) [32–34].

If left unchecked, Ghana’s growing number of drug-resistant cases will make it extremely difficult to keep TB under control. The rise in pre-XDR TB cases highlight the need for proactive and prompt measures, such as ongoing patient monitoring, patient counselling and assistance to increase adherence to treatment, and medication supply management to stop the spread of XDR-TB [35]. In addition to its usage in MTB infection, the indiscriminate use of FQs for treatment of common diseases, such as pneumonia and pyrexia of unknown origin, may be the cause of the increased burden of FQ resistance in pre-XDR-TB patients ([18–36]).

In our study, mutations in *gyrA* and *rrs* genes were responsible for resistance to second-line anti-TB drugs. FQ resistance was due to *gyrA* gene mutation, while injectable second line drug resistance was due to *rrs* gene mutation (AMG). It has been reported that, a mutation in the *gyrA* codon imparts resistance to Levofloxacin and is linked to low-level resistance to Moxifloxacin, while a mutation in the *rrs* gene is linked to high-level resistance to Amikacin, Capreomycin, and Kanamycin [18]. In this study, the *gyrA* mutations were found to occur in codons 90, 91, and 94 with A90V, S91P, D94A, and D94G mutations while the *rrs* mutations found were C1402T, G1484T and position 1484 mutations. The observation of the current study conform with the reports of previous studies from, China, Cambodia, Ethiopia and Ghana [18,19,32,33].

It is noteworthy that codon 94 hosted most of the frequently seen mutations among FQ resistant isolates. Codon 94 substitutions are a phenomenon that may arise because quinolones target the water-magnesium ion bridge with a conserved C3/C4 keto acid moiety, which plays a crucial stabilizing role for the quinolone molecule in the quinolone binding pocket. Therefore an amino acid substitution at this position will exaggerate the detrimental effect of the binding between most quinolones and DNA gyrase [16].

*gyrA* mutations may serve as a potential diagnostic marker for FQ, MDR, and a potential predictor of Pre-XDR-TB or XDR-TB, based on empirical evidence linking *gyrA* mutations to FQ, MDR, Pre-XDR, and XDR-TB [16]. It has been reported that epistasis influences the interaction between rifampicin and FQ-resistant mutations in mycobacterium, which results in variable degrees of fitness loss [37]. The positive epistasis between *gyrA* mutations and mutations in the drug-resistant gene conferring rifampicin resistance may be the cause of the progression of MDR to Pre-XDR or XDR [16]. Hence, it is recommended that more studies be conducted to assess the epistasis between *gyrA* mutations and mutations in drug resistant genes conferring rifampicin resistance for the control of pre-XDR and XDR-TB among the study cohort.

## Conclusion

The *katG (S315T1)* gene mutation accounted for most (73.3%) of the INH resistance, while the predominant *rpoB* gene mutation was S531L (42.47%).

The proportion of pre-XDR-TB among MDR-TB patients in Ghana was slightly higher (7.4%) than that reported in other countries like Ethiopia, but no XDR-TB was detected. The most common fluoroquinolone and aminoglycoside resistance conferring mutations associated with pre-XDR-TB were D94A and C1402T respectively. Sustained surveillance of pre-XDR-TB and XDR-TB is highly recommended.

## Supporting information

S1 Table Meta data of achived isolates showing results of culture, smear and line probe assaya. (PDF)

S2 Table List of MDR isolates showing mutation confering resistance to first and second line TB drugs. (PDF)
