# Supplementary material for: Genotypic analysis of drug-resistant tuberculosis in Ghana: Insights into pre-XDR and XDR-TB
Source: PLoS One. 2025 May 20;20(5):e0323527. doi: 10.1371/journal.pone.0323527 (PMC12091752; doi:10.1371/journal.pone.0323527)
Supplement: S1 Table — (PDF) [file pone.0323527.s001.pdf]

S1 Meta data of archived isolates showing results of culture, smear and Line Probe assays

No	Sex	Region	Culture	MGIT SMEAR	Rif	INH	S	E		Rif	INH	Rif Wild Type Band(s) Absent	Rif Mutation Band(s)	INH Wild Type Band(s) Absent	INH Mutation Band(s)
1	M	Ashanti	MTB	NEGATIVE	R	R	R	R	MTB	R	R(H)	*rpoBWT8*	*rpoBMUT3*	*katGWT*	*KatGMUT1*
2	M	Eastern	MTB	NEGATIVE					MTB	R	R(H)	*rpoBWT3 &4*	*rpoB MUT1*	*katGWT*	
3	M	Eastern	MTB	NEGATIVE					MTB	R	S	*rpoBWT8*			
4	M	Eastern	MTB	NEGATIVE					MTB	R	R(H)	*rpoBWT8*	*rpoB MUT3*	*katGWT*	*KatGMUT1*
5	M	Ashanti	MTB	Positive (Cord)	R	R	S	R	MTB	R	R(H)		*rpoB MUT1*		*KatGMUT1*
6	M	Eastern	MTB	NEGATIVE					MTB	R	R(H)	*rpoBWT3&4*	*rpoB MUT1*	*katGWT*	*katG MUT1*
7	M	Ashanti	MTB	NEGATIVE					MTB	R	R(H)	*rpoBWT8*	*rpoBMUT3*		*KatGMUT1*
8	M	Ashanti	MTB	NEGATIVE					MTB	R	R(H)	*rpoBWT7*	*rpoB MUT2B*	*katGWT*	
9	M	Eastern	MTB	NEGATIVE	R	R	R	R	MTB	R	S		*rpoB MUT3*		
10	M	Ashanti	MTB	NEGATIVE	R	S	R	R	MTB	R	S	*rpoBWT7*			
11	M	Eastern	MTB	Positive (Cord)	R	R	R	R	MTB	R	S		*rpoB MUT3*		
12	M	Ashanti	MTB	NEGATIVE	R	S	R	R	MTB	R	R(H)		*rpoB MUT3*	*katGWT*	*katG MUT1*
13	M	Eastern	MTB	NEGATIVE	R	S	R	R	MTB	R	R(H)	*rpoBWT8*	*rpoB MUT3*	*katGWT*	*katG MUT2*
14	F	Ashanti	MTB	NEGATIVE	R	R	R	R	MTB	R	R(H)		*rpoBMUT3*		*KatGMUT1*
15	F	Eastern	MTB	Positive (Cord)	R	S	R	R	MTB	R	S	*rpoBWT8*			
16	M	Eastern	MTB	Positive (Cord)					MTB	R	S	*rpoBWT8*			

17	M	Ashanti	MTB	NEGATIVE					MTB	R	S	*rpoBWT8*	*rpoBMUT3*		
18	M	Eastern	MTB	Positive (Cord)					MTB	R	R(L)	*rpoBWT8*	*rpoBMUT3*		*inhAMUT1*
19	M	Eastern	MTB	NEGATIVE	R	R	R	R	MTB	R	S	*rpoBWT3 &4*			
20	M	Ashanti	MTB						MTB	R	R(H)	*rpoBWT8*	*rpoB MUT3*	*katGWT*	*katG MUT1*
21	M	Eastern	MTB	Positive (Cord)					MTB	R	S	*rpoBWT8*			
22	M	Eastern	MTB	Positive (Cord)					MTB	R	R(H)	*rpoBWT8*	*rpoB MUT3*	*katGWT*	*katG MUT1*
23	M	Eastern	MTB						MTB	R	R(H)	*rpoBWT8*	*rpoB MUT3*	*katGWT, inhA WT2*	*katG MUT1, inhA MUT3A*
24	F	Eastern	MTB						MTB	R	R(H)	*rpoBWT8*	*rpoB MUT3*	*katGWT*	*katG MUT1*
25	M	Ashanti	No Growth						MTB	R	S	*rpoBWT8*	*rpoBMUT3*		
26	M	Ashanti	MTB						MTB	R	S	*rpoBWT8*	*rpoB MUT3*		
27	M	Eastern	MTB						MTB	R	R(H)	*rpoBWT7*	*rpoBMUT2A*		*KatG MUT1*
28	M	Ashanti	MTB	Positive (Cord)					MTB	R	R(H)		*rpoBMUT2A*		*KatG MUT1*
29	M	Eastern	MTB						MTB	R	S	*rpoBWT8*	*rpoB MUT3*		*inhAMUT1*
30	M	Central	MTB	Positive (Cord)	R	R	R	S	MTB	R	R(H)	*rpoBWT3 &4*	*rpoB MUT1*	*katGWT*	*KatG MUT2*
31	M	Volta	MTB	Positive (Cord)					MTB	R	S	*rpoBWT8*			
32	F	Eastern	MTB	Positive (Cord)	R	S	S	R	MTB	R	R	*rpoBWT3 &4*		*katGWT*	*katG MUT2*
33	M	G. Accra	MTB						MTB	R	R(H)		*rpoB MUT2B*	*katGWT*	*KatGMUT1*

34	M	Central	MTB	Positive (Cord)	R	R	S	S	MTB	R	S	*rpoBWT7*			
35	F	Central	MTB	Positive (Cord)	R	R	R	S	MTB	R	R(H)	*rpoBWT8*	*rpoB MUT3*		*katG MUT1*
36	M	G. Accra	MTB	Positive (Cord)	R	S	S	S	MTB	R	R(L)	*rpoBWT8*	*rpoB MUT3*		*inhA MUT1*
37	F	Central	MTB	Positive (Cord)	R	S	S	S	MTB	R	S		*rpoB MUT2B*		
38	M	Ashanti	MTB						MTB	R	R(H)	*rpoBWT3&4*	*rpoB MUT1*		*katG MUT1*
39	M	Eastern	MTB						MTB	R	R(H)	*rpoBWT3&4*			*KatGMUT1*
40	F	Central	MTB	Positive (Cord)					MTB	R	R(H)	*rpoBWT3&4*	*rpoB MUT1*	*KatGWT*	*KatGMUT1*
42	M	Eastern	MTB	Positive (Cord)	R	R	R	R	MTB	R	R(H)	*rpoBWT8*	*rpoB MUT3*	*KatGWT*	*KatGMUT1*
42	M	Central	MTB	Positive (Cord)	R	R	R	S	MTB	R	S	*rpoBWT8*	*rpoB MUT2B*		
43	M	Central	MTB	NEGATIVE	R	S	R	S	MTB	R	S	*rpoBWT8*	*rpoB MUT3*		
44	M	Central	MTB	Positive (Cord)	R	R	R	R	MTB	R	R(H)	*rpoBWT3&4*	*rpoB MUT1*	*KatGWT*	*KatGMUT1*
45	M	Eastern	MTB	Positive (Cord)	R	R	R	R	MTB	R	S	*rpoBWT8*	*rpoB MUT3*	*katGWT*	
46	M	Eastern	MTB						MTB	R	R(H)		*rpoB MUT2B*	*katGWT*	*katG MUT1*
48	M	Eastern	MTB						MTB	R	R(H)	*rpoBWT3&4*	*rpoB MUT1*	*katGWT*	*katG MUT1*
48	M	Central	MTB						MTB	R	R(L)	*rpoB WT8*	*rpoB MUT3*	*inhAWT1*	
49	M	Central	MTB						MTB	R	R(H)	*rpoB WT8*	*rpoB MUT3*	*katGWT*	*katG MUT1*
50	M	Eastern	MTB						MTB	R	S		*rpoB MUT3*		
51	M	Eastern	MTB						MTB	R	R(L)		*rpoB MUT2B*	*inhAWT1*	

52	M	Eastern	MTB						MTB	R	R(H)		*rpoB MUT2B*	*katGWT*	
53	M	Eastern	MTB						MTB	R	R(H)	*rpoBWT3&4*	*rpoB MUT1*	*katGWT*	*katG MUT1*
54	M	Eastern	MTB						MTB	R	S	*rpoBWT1*			*katG MUT1*
55	M	Eastern	MTB						MTB	R	R(H)	*rpoBWT8*		*katGWT*	*katG MUT1*
56	M	Eastern	No Growth						MTB	R	R(L)	*rpoBWT8*	*rpoBMUT3*	*inhAWT1*	
57	F	Eastern	MTB						MTB	R	R(H)	*rpoBWT8*	*rpoB MUT3*	*katGWT*	*katG MUT1*
58	M	Eastern	No Growth						MTB	R	R(H)	*rpoBWT2&3*		*katGWT*	*katGMUT1*
59	M	Eastern	MTB	Positive (Cord)	R	S	R	R	MTB	R	S	*rpoBWT8*	*rpoB MUT3*		
60	M	Western	MTB						MTB	R	S	*rpoBWT8*			
61	M	G. Accra	MTB						MTB	R	S		*rpoBMUT3*		
62	M	G. Accra	MTB						MTB	R	S	*rpoBWT7*	*rpoBMUT2A*		
63	M	Western	MTB						MTB	R	R(H)	*rpoBWT7*	*rpoBMUT2 B*	*katGWT*	*katGMUT1*
64	M	Upper West	MTB						MTB	R	S	*rpoBWT8*			
65	M	G. Accra	MTB						MTB	R	R(H)	*rpoBWT7*		*katGWT*	*katGMUT1*
66	F	G. Accra	MTB						MTB	R	S	*rpoBWT8*	*rpoBMUT3*		
67	M	G. Accra	MTB						MTB	R	R(H)	*rpoBWT3&4*	*rpoBMUT1*	*katGWT*	*katGMUT1*
68	M	G. Accra	MTB						MTB	R	S	*rpoBWT7*	*rpoBMUT2A*		

69	M	G. Accra	MTB						MTB	R	S	*rpoBWT8*			
70	M	G. Accra	MTB						MTB	R	R(H)		*rpoBMUT2B*	*katGWT*	*KatGMUT1*
71	F	Western	MTB						MTB	R	S	*rpoBWT8*	*rpoBMUT3*		
72	M	G. Accra	MTB						MTB	R	R(H)	*rpoBWT8*	*rpoBMUT3*	*KatGWT*	*KatGMUT1*
73	M	Western	MTB						MTB	R	R(H)	*rpoBWT7*	*rpoBMUT2A*	*katGWT*	*KatGMUT1*
