# Supplementary material for: Genotypic analysis of drug-resistant tuberculosis in Ghana: Insights into pre-XDR and XDR-TB
Source: PLoS One. 2025 May 20;20(5):e0323527. doi: 10.1371/journal.pone.0323527 (PMC12091752; doi:10.1371/journal.pone.0323527)
Supplement: S2 Table — (PDF) [file pone.0323527.s002.pdf]

S2: LIST OF MDR ISOLATES SHOWING MUTATIONS CONFERRING RESISTANCE TO FIRST- AND SECOND-LINE TB DRUGS

NO	Culture Results	RIF. Mutations	INH Mutations	FQs Mutations	AMGs Mutations	Genotypic Results - First Line	Genotypic Results - Second line
1	MDR-TB	S531L	S315T1			MDR-TB	
2	MDR-TB	D516V	at codon 315			MDR-TB	
3	MDR-TB	at Codon 530-533				Rif Resistance Inferred.	
4	MDR-TB	S531L	S315T1			MDR-TB	
5	MDR-TB	F505L	S315T1			MDR-TB	
6	MDR-TB	D516V	S315T1			MDR-TB	
7	MDR-TB	S531L	S315T1			MDR-TB	
8	MDR-TB	H526D	at codon 315			MDR-TB	
9	MDR-TB	S531L		**D94A**		RR-MTB	Pre-XDR-TB
10	MDR-TB	at codons 526-529				Rif Resistance Inferred.	
11	MDR-TB	S531L				RR-MTB	
12	MDR-TB	S531L	S315T1	**D94G**		MDR-TB	Pre-XDR-TB
13	MDR-TB	S531L	S315T2			MDR-TB	
14	MDR-TB	S531L	S315T1		**C1402T**	MDR-TB	Pre-XDR-TB
15	MDR-TB	at codon 530-533			**at 1484 region**	Rif Resistance Inferred.	AMG resistance inferred
16	MDR-TB	at codon 530-533		**D94A**		Rif Resistance Inferred.	Pre-XDR-TB
17	MDR-TB						
18	MDR-TB						
19	MDR-TB	S531L			**C1402T**	RR-MTB	Pre-XDR-TB
20	MDR-TB						

21	MDR-TB	S531L	C-15t			MDR-TB	
22	MDR-TB	at codons 510-517				Rif Resistance Inferred	
23	MDR-TB	S531L	S315T1			MDR-TB	
24	MDR-TB						
25	MDR-TB	at codon 530-533				Rif Resistance Inferred.	
26	MDR-TB	S531L	S315T1			MDR-TB	
27	MDR-TB	S531L	S315T1, t-8c			MDR-TB	
28	MDR-TB	S531L	S315T1			MDR-TB	
29	MDR-TB						
30	MDR-TB						
31	MDR-TB	S531L			**G1484T**	RR-MTB	Pre-XDR-TB
32	MDR-TB	H526Y	S315T1			MDR-TB	
33	MDR-TB	H526Y	S315T1			MDR-TB	
34	MDR-TB	at codons 526-529				Rif Resistance Inferred	
35	MDR-TB	at codon 530-533			**at position 1484**	Rif Resistance Inferred.	AMG Resistance inferred
36	MDR-TB	at codons 526-529				Rif Resistance Inferred	
37	MDR-TB	D516V	S315T2			MDR-TB	
38	MDR-TB	D516Y at codons 513-519		**S91P**		Rif Resistance Inferred.	Pre-XDR-TB
39	MDR-TB	H526D	S315T1			MDR-TB	
40	MDR-TB	S531L	S315T1			MDR-TB	
41	MDR-TB	S531L	C–15T			MDR-TB	
42	MDR-TB	H526D				RR-MTB	
43	MDR-TB	D516V	S315T1			MDR-TB	

44	MDR-TB	D516Y at codons 513-519	S315T1			MDR-TB	
45	MDR-TB	D516V	S315T1	**D94A**		MDR-TB	Pre-XDR-TB
46	MDR-TB	S531L	S315T1			MDR-TB	
47	MDR-TB	H526D			**C1402T**	RR-MTB	Pre-XDR-TB
48	MDR-TB	S531L				RR-MTB	
49	MDR-TB	D516V	S315T1			MDR-TB	
50	MDR-TB	S531L	at codon 315			MDR-TB	
51	MDR-TB	H526D	S315T1			MDR-TB	
52	MDR-TB						
53	MDR-TB						
54	MDR-TB	D516V	S315T1			MDR-TB	
55	MDR-TB	S531L	in the -15 region			MDR-TB	
56	MDR-TB	S531L	S315T1	**A90V**		MDR-TB	Pre-XDR-TB
57	MDR-TB						
58	MDR-TB	S531L				RR-MTB	
59	MDR-TB	H526D	in the -15 regiond			MDR-TB	
60	MDR-TB	H526D	at codon 315			MDR-TB	
61	MDR-TB						
62	MDR-TB	S531L				RR-MTB	
63	MDR-TB	S531L				RR-MTB	
64	MDR-TB	D516V	S315T1			MDR-TB	
65	MDR-TB	at codons 505-509	S315T1			MDR-TB	
66	MDR-TB						
67	MDR-TB	at codon 530-533	S315T1			MDR-TB	
68	MDR-TB	S531L	S315T1			MDR-TB	

69	MDR-TB	S531L				RR-MTB	
70	MDR-TB	at codon 530-533				RR-MTB	
71	MDR-TB	H52Y				RR-MTB	
72	MDR-TB	H52D	S315T1			MDR-TB	
73	MDR-TB	at codon 530-533				Rif Resistance Inferred	
74	MDR-TB	at codon 526-529	S315T1			MDR-TB	
75	MDR-TB	D516V	S315T1			MDR-TB	
76	MDR-TB	H526Y				RR-MTB	
77	MDR-TB	at codon 530-533				RR-MTB	
78	MDR-TB	H526D	S315T1			MDR-TB	
79	MDR-TB	S531L				RR-MTB	
80	MDR-TB	S531L	S315T1			MDR-TB	
81	MDR-TB	H526Y	S315T1			MDR-TB	
